# The Extrachromosomal DNA Fusion Factory: Extrachromosomal Genesis of Oncogenic Fusion Transcripts

**DOI:** 10.34133/research.1291

**Published:** 2026-05-27

**Authors:** Yasen Maimaitiyiming, Chang Yang, Hua Naranmandura

**Affiliations:** ^1^ Department of Immunology and Institute of Basic Medical Sciences, School of Basic Medical Sciences, Xinjiang Medical University, Urumqi 830011, China.; ^2^ Xinjiang Key Laboratory of Molecular Biology for Endemic Diseases, Xinjiang Medical University, Urumqi 830011, China.; ^3^ Department of Hematology of First Affiliated Hospital, Zhejiang University School of Medicine, Hangzhou 310058, China.; ^4^ Department of Public Health, Zhejiang University School of Medicine, Hangzhou 310058, China.

## Abstract

Fusion oncogenes are among the most potent and clinically actionable cancer drivers, contributing to approximately 15% to 20% of human malignancies. Traditionally viewed as stable consequences of chromosomal translocations, fusion oncogenesis has long been interpreted through a DNA-centric paradigm. However, this view is now challenged by the discovery of a vast and dynamic landscape of fusion RNAs. Recent integrative genomic studies suggest that extrachromosomal DNA (ecDNA) can serve as a platform for the formation, diversification, and amplification of oncogenic fusion transcripts in multiple cancer contexts. Here, we propose the “ecDNA fusion factory” as a unifying conceptual framework that integrates nonchromosomal genome architecture, structural instability, and RNA-level regulatory consequences to expand current understanding about the origin and adaptive evolution of some fusion drivers. In this model, fusion output can be dynamic and heterogeneous, shaped by ecDNA copy-number flux and context-dependent structural remodeling rather than exclusively by a single, fixed chromosomal breakpoint. This reframing suggests distinct therapeutic vulnerabilities, potentially motivating strategies that target both the fusion oncoprotein and the ecDNA platform that sustains it, and necessitates a revision of diagnostic paradigms to detect these structurally plastic and dynamically evolving oncogenic elements. Together, this perspective broadens current models of fusion-driven oncogenesis and highlights possible implications for precision oncology.

## Background

From the discovery of *BCR::ABL* in chronic myeloid leukemia to *PML::RARA* in acute promyelocytic leukemia and *EML4::ALK* in lung cancer, fusion oncogenes have served as definitive diagnostic markers, dominant tumor drivers, and paradigms for targeted therapy [1,2]. Pan-cancer analyses indicate that approximately 15% to 20% of human malignancies harbor driver gene fusions [3,4]. Their prevalence is exceptionally high in certain hematologic malignancies, such as chronic myeloid leukemia and acute promyelocytic leukemia, and can be particularly high in select solid tumor contexts (e.g., many sarcomas and subsets of prostate cancers) [1–4]. The clinical success of small molecules targeting fusion-driven oncoproteins—including BCR-ABL, PML-RARA, EML4-ALK, ROS1, RET, and NTRK—underscores the enduring translational importance of fusion biology [1,2,4,5].

**Fig. 1.**
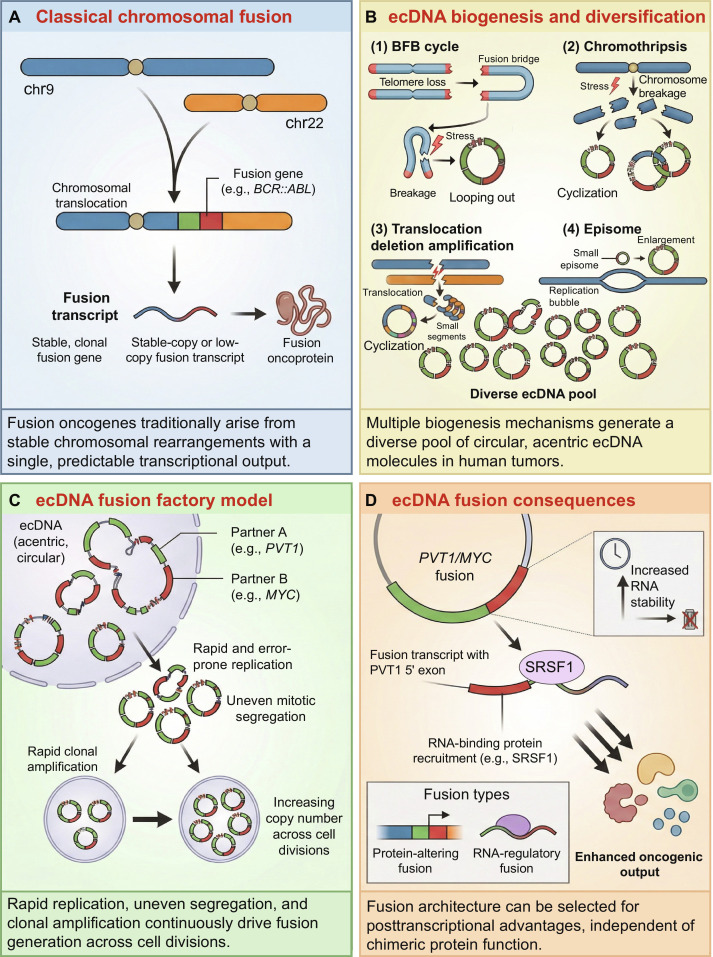
The extrachromosomal DNA (ecDNA) fusion factory: a dynamic platform for oncogenic fusion transcript generation and evolution. (A) Classical chromosomal fusion paradigm. Chromosomal translocations (e.g., between Chr9 and Chr22) produce a stable, clonal fusion gene with predictable transcriptional output. (B) EcDNA biogenesis and diversification. Multiple mechanisms—including breakage–fusion–bridge (BFB) cycles, chromothripsis, translocation-coupled episome formation, and small episome replication bubbles—generate a diverse pool of circular, acentric ecDNA molecules. (C) EcDNA fusion factory model. EcDNAs may carry partner genes (e.g., PVT1 and MYC). Rapid, error-prone replication and uneven mitotic segregation lead to rapid clonal amplification and increasing copy number across cell divisions, creating opportunities for structural remodeling and, in some contexts, the emergence of novel fusion configurations. (D) ecDNA fusion consequences. Fusion transcripts can confer selective advantages through multiple mechanisms, including RNA-binding protein recruitment (e.g., serine/arginine-rich splicing factor 1 [SRSF1] enhancing *PVT1::MYC* RNA stability), protein-altering fusions, and RNA-regulatory fusions—all of which enhance oncogenic output independently of a fixed chromosomal breakpoint.

Historically, fusion oncogenes have been conceptualized through a chromosome-centric framework, in which chromosomal translocations or inversions generate stable fusion genes that are clonally inherited and faithfully transcribed into oncogenic chimeric RNAs (Fig. 1). This model has been particularly effective in hematologic malignancies, where recurrent rearrangements are readily detected by cytogenetics and fluorescence in situ hybridization (FISH) [1,2]. However, the very success of these DNA-based assays may have obscured other nonchromosomal sources of fusion transcripts.

High-throughput RNA sequencing has revealed a much broader landscape of fusion transcripts across tumor types, many of which lack an obvious recurrent DNA-level correlate [3,6,7]. Proposed mechanisms include readthrough transcription, *cis*- and *trans*-splicing, and other RNA-processing events [6,7]. While these mechanisms likely explain part of the observed fusion transcriptome, they do not fully account for recurrent, highly expressed, and apparently selected fusion transcripts in all contexts.

## Extrachromosomal DNA: an architecturally permissive substrate

Extrachromosomal DNA (ecDNA) provides a compelling substrate to reconcile these observations. EcDNA can arise through multiple biogenesis mechanisms, including breakage–fusion–bridge cycles, chromothripsis, and episome formation [8,9] (Fig. 1). These processes generate circular, acentric DNA molecules that lack centromeres and replicate autonomously. Unlike chromosomal DNA, ecDNA replicates asynchronously, segregates unevenly during mitosis, and undergoes extensive structural rearrangement [9,10]. Across cancer types, ecDNA is strongly associated with intratumoral heterogeneity, aggressive disease, and poor clinical outcome [8,10,11].

Accumulating integrative genome-transcriptome analyses suggest that ecDNA harbors a disproportionately high burden of structural variants and highly expressed oncogenic fusion transcripts [12]. These findings position ecDNA not merely as a passive amplifier but as a plausible and potentially active substrate for fusion generation, amplification, and context-dependent selection of fusion-associated outputs—a “fusion factory” in which circular genome architecture, structural instability, and variable copy number converge to drive oncogenic output (Fig. 1).

## EcDNA may provide a dynamic substrate for fusion transcript generation and selection

Within the ecDNA factory, fusion generation may not be restricted to a single initiating event and, in some contexts, could continue through ecDNA structural remodeling over time. The circular architecture, atypical replication dynamics, and acentric nature of ecDNA are associated with DNA damage, uneven segregation, and reliance on error-prone repair pathways. Kang et al. [13] show that ecDNA replication introduces additional DNA entanglements and torsional stress, leading to the generation of abortive topoisomerase cleavage complexes that activate the ataxia-telangiectasia mutated-mediated DNA damage response. The resulting double-strand breaks are repaired primarily by the alternative nonhomologous end-joining pathway involving POLθ and LIG3. This provides a mechanistic foundation for recurrent fusion formation on ecDNA.

However, whether such structural reshuffling occurs continuously under basal, unstimulated conditions or is primarily triggered by replication stress, DNA damage, or therapy remains an open question. Nonetheless, the high baseline burden of ecDNA-borne structural variants suggests that fusion generation can occur even without overt external selection, though it remains unclear whether this reflects continuous low-level reshuffling or sporadic, event-driven processes [12,13]. Uneven segregation of ecDNA during mitosis then rapidly diversifies ecDNA content and dosage across tumor cells, embedding them in a dynamic evolutionary landscape where selection can act on the functional impact of the fusion transcript itself [12,14].

Critically, in some contexts, selective advantage may arise from RNA-level regulatory properties of the fusion transcript, rather than solely from its protein-coding output. The landmark study by Yi et al. [12] demonstrates this principle through the recurrent PVT1/MYC ecDNA amplicon in which the fusion architecture recruits serine/arginine-rich splicing factor 1 to enhance RNA stability and oncogenic output. This work provides strong evidence that RNA stability can be a selectable and evolvable trait within the ecDNA context. However, whether RNA-centric selection represents a universal feature of ecDNA-borne fusions or is restricted to specific genomic and cellular contexts remains an open question. Other ecDNA-driven oncogenic variants, such as epidermal growth factor receptor variant type III in glioblastoma, may confer selective advantage primarily through enhanced protein signaling and stronger clonal fitness, rather than RNA-level mechanisms [15]. Future studies should therefore determine the spectrum of regulatory mechanisms by which ecDNA confers selective advantage and assess whether classic fusion oncogenes (e.g., *BCR::ABL* and *PML::RARA*) can also be amplified, remodeled, or, in some cases, generated through this dynamic platform.

## EcDNA-fusion synergy drives aggressive phenotypes and therapeutic evolution

The convergence of ecDNA amplification and fusion oncogenes may define a powerful oncogenic circuit with direct clinical implications. EcDNA supplies extreme copy-number flux and structural plasticity, while fusion oncogenes deliver potent, lineage-defining driver functions [12]. Together, these features may generate tumors characterized by high transcriptional output, rapid adaptability, and pronounced heterogeneity, which may underlie particularly aggressive clinical phenotypes [12,13,16].

This synergy also offers a conceptual framework for understanding therapeutic resistance. Therapy can act as a selective pressure, driving the expansion of ecDNA clones harboring resistance-conferring fusion variants or novel, therapy-escaping configurations. While therapy-induced DNA damage may promote ecDNA formation [16], recent evidence suggests that nascent ecDNA molecules can undergo structural rearrangement and, in some contexts, give rise to novel fusion configurations [12]. Consequently, resistance can emerge not only through sequence mutations but also via extrachromosomal pathways: (a) structural remodeling/amplification of fusion-associated transcripts, (b) dynamic isoform switching, or (c) ecDNA-driven transcriptional rewiring. These plastic and dosage-driven mechanisms evade conventional assays designed for static chromosomal fusions.

## Therapeutic and diagnostic implications of the ecDNA fusion model

This paradigm supports a reassessment of both therapeutic and diagnostic strategies [17,18]. Therapeutically, effective intervention may require dual-pronged approaches: (a) disrupting the maintenance, replication, or segregation of ecDNA [19,20]; and (b) exploiting ecDNA-specific outputs, such as enhanced RNA stability, aberrant transcriptional condensates, or novel fusion-derived neoantigens [12].

Diagnostically, the dynamic, nonclonal, and RNA-centric nature of ecDNA-borne fusions challenges standard assays such as FISH or targeted PCR, which are highly effective for detecting known, recurrent chromosomal rearrangements. FISH is generally optimized for stable, known chromosomal translocations and is therefore less suited to detecting ecDNA-borne fusions with unknown joining points or sequence configurations. Likewise, PCR across a known genomic breakpoint may miss fusions that arise through structural remodeling of ecDNA or transcripts whose expression is heterogeneous, unstable, or not linked to a single canonical chromosomal breakpoint. These limitations may contribute to a subset of clinically aggressive but “fusion-negative” tumors. Integrating RNA sequencing and long-read DNA sequencing into diagnostic workflows, particularly for therapy-resistant cancers with known ecDNA prevalence, may be important to uncover the full oncogenic repertoire and guide precision treatment.

## Outlook

In conclusion, recognizing ecDNA as a potential source, amplifier, and diversifier of oncogenic fusion transcripts broadens current models of fusion oncogenesis. The ecDNA fusion factory model integrates structural genome instability with RNA-level regulation into a dynamic evolutionary framework, suggesting an additional layer of molecular plasticity in cancer. Recognizing this plasticity may encourage the development of adaptive diagnostics and innovative therapies that target both the products of the ecDNA factory and the factory itself. Defining the prevalence, mechanisms, and clinical relevance of ecDNA-driven fusions across cancer types will be essential to delineate the full impact of this emerging paradigm. Moving forward, oncology may need to expand beyond a static, chromosomal view of fusions to a broader framework that also incorporates dynamic extrachromosomal mechanisms. Recognizing tumors as ecosystems containing evolving “fusion factories” is pivotal for developing adaptive therapies and overcoming the formidable challenge of cancer evolution.
